# Concept, hardware development, and clinical trials of a Galinstan based Mercury free sphygmomanometer: Merkfree

**DOI:** 10.1038/s41598-022-19926-7

**Published:** 2022-09-22

**Authors:** Ravinder Kumar, Mohit Kumar, Gurpreet Singh Wander, Ashish Kumar Sahani

**Affiliations:** 1 Department of Biomedical Engineering, Indian Institute of Technology, Ropar, India; 2 Dayanand Medical College and Hospital (DMC&H), Ludhiana, India

**Keywords:** Health care, Public health, Biomedical engineering

## Abstract

The aim of this work is to develop Merkfree—a mercury-free sphygmomanometer that looks, feels, and operates just like a traditional mercury sphygmomanometer (MS). For this we use Galinstan as a substitute for mercury, which is a non-toxic alloy of Gallium, Indium and Tin. Galinstan is nearly half as dense as mercury and sticks to class. To work with the lower density, we designed an enclosure and scale that is nearly double the length of MS. The issue of stickiness with glass was resolved by maintaining a small meniscus of a reducing agent in the measuring tube and tank of Merkfree. Clinical trials to validate the accuracy of Merkfree against MS and oscillometric sphygmomanometer (OS) were conducted over 252 patients. The results show a good correlation of the systolic and diastolic BP measurements from Merkfree with respect to MS and the OS. The mean absolute percentage error is less than 10% for both SBP and DBP. We also found that Merkfree has lower rounding-off errors compared to MS. Merkfree can be a viable alternative to mercury sphygmomanometer that can help achieve the goal of WHO in eliminating mercury from healthcare, while simultaneously making sure that gold standard technique of sphygmomanometry continues to be available to the clinicians.

## Introduction

Globally, over 1.3 billion people live with hypertension, and it is the commonest non communicable disease. It is a major risk factor for cardiovascular diseases (CVD). Around 18.6 million lives every year are affected by CVD^1^. Accurate measurement of BP is important to make sure that those who require medications are not missed and unnecessary medication use is avoided^2^. BP monitors are required in the outpatient and in-patient areas across all medical specialties. The three most widely accepted BP measurement devices are the MS, the aneroid sphygmomanometer (AS) and the Oscillometric sphygmomanometer (OS)^3,4^. MS is considered as the gold standard in BP measurement. It is based on Bernoulli’s principle and uses the auscultatory method. The height of mercury column is the pressure gauge indicator. The pressure indicated is a function of the density and height of the mercury column. Density, a physical property of mercury, ensures that the readings are reliable even under low maintenance conditions. In comparison, the aneroid type sufferers from degrading accuracy with time as its spring loosens with use. The Oscillometric devices, measure the mean pressure. The systolic and diastolic pressures are derived using proprietary algorithms that vary from company to company and are primarily maintained as trade secrets. For this reason, there are issues regarding reliability of devices available in low and middle-income countries (LMICs) where the prevalence of hypertension is increasing^5–11^. Owing to established standard procedures, the inertia of change and accuracy issues, the clinician community does not place in these methods the same faith as the MS^12^. In an anonymous survey of 138 medical practitioners conducted by us in India, 82% prefer MS over AS and OS. 49% felt MS is the most robust and has least breakdown. 88% would prefer a manual apparatus like the MS over the AS and OS for office use^13^. According to recent India heart study conducted by us, till today, 70% of Indian clinicians use the MS^14^. Thus, there is an unmet need in the market for a mercury-free sphygmomanometer with the same structure and principle of operation as the MS after the mercury ban due to Minamata convention comes into effect. The mercury device is being banned not because of the invention of any technology of BP measurement but because of the harmful effects of mercury. India is a signatory to the Minamata Convention undertaking and is obliged to eliminate use of mercury-based products in medical equipment in the next couple of years^15^. Galinstan is a promising substitute for mercury. It is a non-toxic alloy of Gallium, Indium and Tin and its density is about half that of the mercury, and a melting point of approximately − 19 °C^16^. Galinstan is being used in various areas of applications such as in microdevices^17^, actuators, electrochemistry, 3D patterning, microfluidics, elastomeric composites, soft electronics^16^, integrated liquid cooling system^18^, and generation of electrically conductive composites for electronic applications. There are some other eutectic gallium indium based alloys such as EGaIn which are liquid at room temperature but Galinstan has higher density and lower melting point as compared to them^19^. Although Galinstan based thermometers are available, there has been very limited attempts to develop a Galinstan based sphygmomanometer. Thermometers are sealed hermetically under vacuum, while in the case of a Sphygmomanometer, one end of the tube must be open to air, to measure gauge pressure. One of the primary reasons is that whenever the material is exposed to oxygen in the air, a layer of gallium oxide is formed, which leads to the stickiness of Galinstan with glass^20^. There is evidence in the literature that surface tension decreases significantly when oxygen is present in liquid metals^21^. Galinstan (GaInSn) stickiness can occur in two modes. The first mode occurs when the oxide shell is not ruptured as it makes contact with the substrate. It occurs due to the nanoscale topology of the oxide surface, regardless of surface energy or substrate texture. Therefore, this mode results in minimal adhesion between the liquid metal and most solids. In the second mode, the GaInSn-substrate interface is formed by rupturing the original oxide skin and creating a composite interface that includes contact between the substrate and pieces of old oxide, bare liquid metal, and new oxide. The formed metal oxide exposed to air produces large yield stress^22^. However various methods have been proposed in recent years to mitigate this issue. Among them, chemical etching with acid (for e.g. HCl) or treatment with base (NaOH) is a widely accepted effective method to eliminate the oxide skin^23^. Moreover, a few researchers have explored the electrochemical process whereby controlling the electrochemical potential on the surface of the liquid metal in an electrolyte quickly and reversibly changes the interfacial tension by over two orders of magnitude^24^. We have designed a mercury free sphygmomanometer and have called it ‘Merkfree’. It uses Galinstan as a measuring fluid and NaOH as an oxide removal agent. To verify its accuracy and usability, we have carried clinical trials which are presented here.

## Materials and methods

### Device design

**Figure 1:**
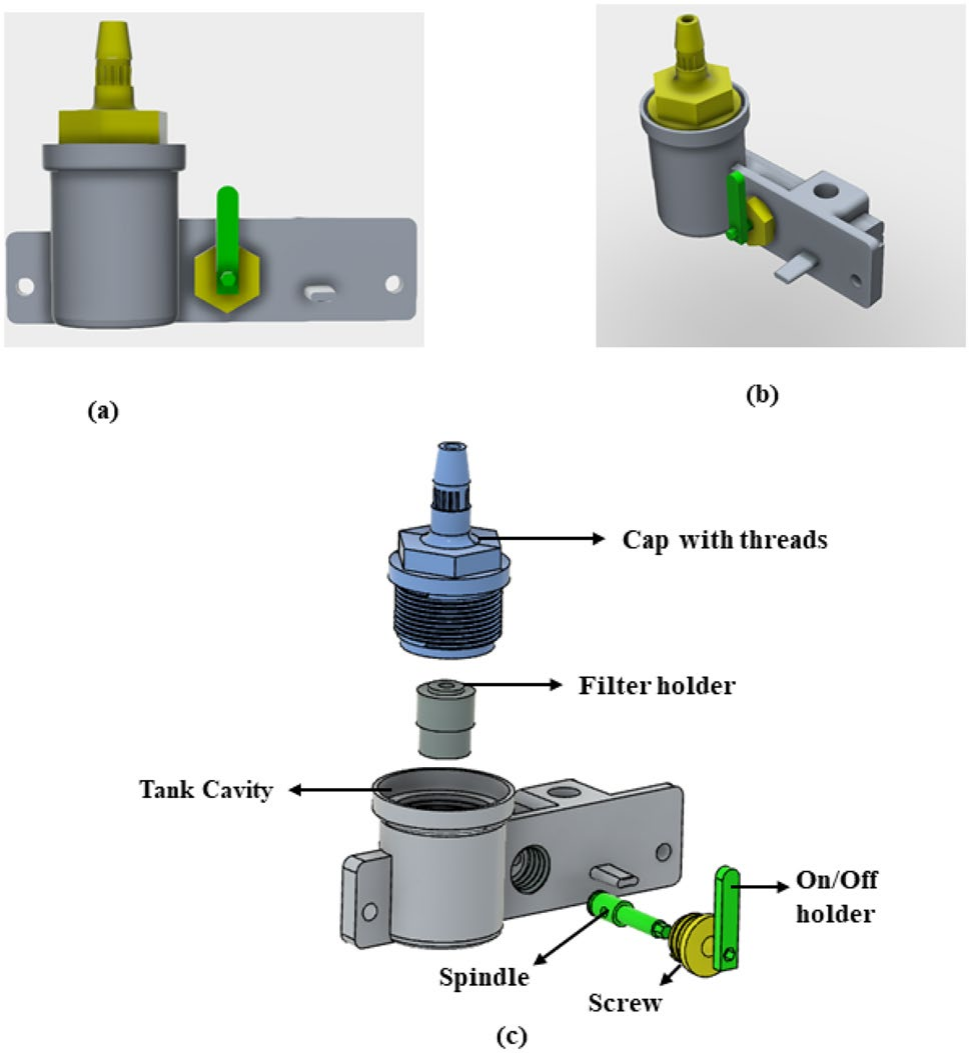
The Galinstan tank with the filter holder and its connection with measuring scale. (**a**) Front 3D design view. (**b**) Isometric 3D design view. (**c**) Exploded view of the full tank.

The Sphygmomanometer was first designed in solid works (CAD designing software). All attributes such as density of measuring fluid, volume requirement of fluid in tank and scale were taken into consideration. The enclosure and the tank were manufactured by 3D printing. The enclosure has been printed by fused deposition modelling (FDM) in which poly lactic acid (PLA) filament was used. The tank and the upper tube holder were printed with stereo lithography assembly (SLA). The device components are explained below. The filter unit contains filter paper which allows air to pass through but doesn’t allow the measuring fluid to get out from it. It also holds the upper side of the glass tube in place. The tank is where Galinstan is stored when device is not in use. This needs to be air-tight to hold air pressure Fig. 1. One end of the tank is connected with the rubber tube from the bulb and the other end is connected with the lower end of the glass tube. The scale was 3D printed by using dual colour filament using FDM technique. To display the measurement in mmHg the scale needs to be recalibrated using the formula shown in Eq. (1)^25^.

1 $$ \Delta H_{tu} = 133.3/\left( {\rho_{m} g\left( {\pi r_{tu}^{2} /A_{tn} + 1} \right)} \right. $$

The $\Delta H_{tu}$ is the change in height of the fluid inside glass tube for every mmHg pressure, $A_{tn}$ is the area of cross-section of the tank, $r_{tu}$ is the inner radius of the glass tube, $\rho_{m}$ is the density of the material. The display scale is calibrated to show the measurement in mmHg since this is the standard measure in all BP sphygmomanometers. Galinstan has a density of 6.44 g/cc which is almost half that of mercury (13.6 g/cc), the column height is nearly doubled. We use a tank with cross-sectional area of 695.6 mm^2^ and a measuring tube with an inner diameter of 3 mm. Thus, by using Eq. (1), the scale is calibrated to 1 mmHg per 2.1 mm.

**Figure 2:**
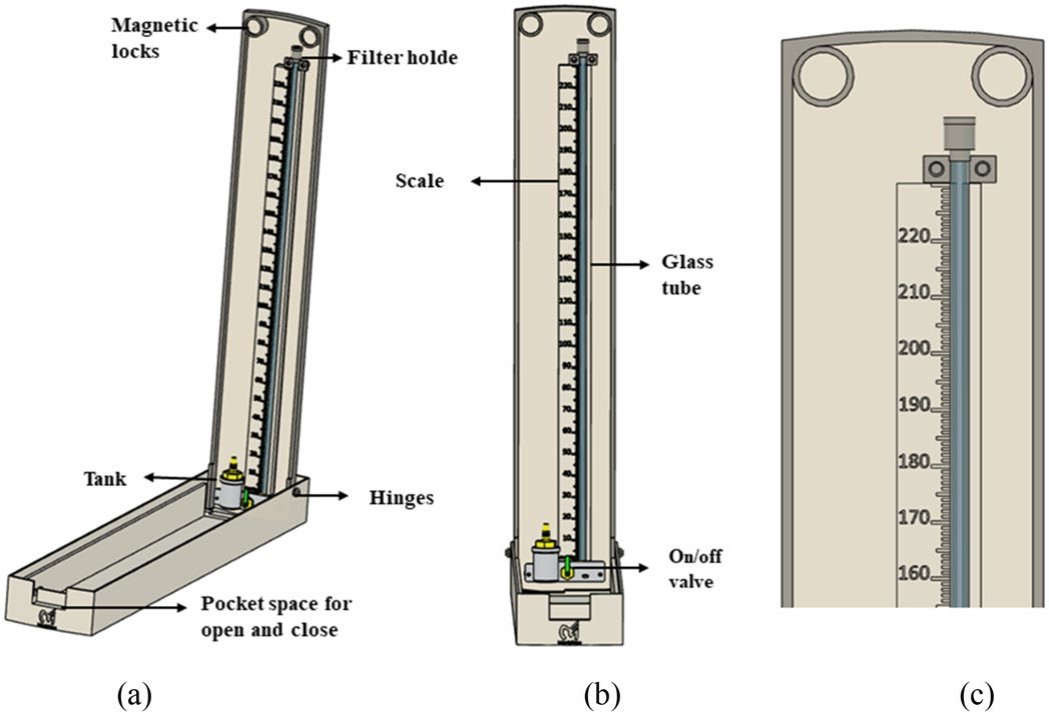
The CAD design of the Merkfree device. (**a**) Side view, (**b**) front view, (**c**) Inset view of the measuring scale and the tube.

The outer enclosure box has two components—the flap on which the tank, and the scale have been attached, and the base box, where all the accessories such as the cuff and the bulb are kept. The flap is connected with the base-box through 3D printed hinges to provide 0-to-120-degree freedom of movement. Magnetic locks were provided in the device between the flap and box as shown in Fig. 2.

### Chemical treatment

Galinstan has Gallium in it, which forms a thin oxide layer when it is exposed to the environment. The thin oxide layer creates a low surface tension and make it stick with the glass tube, disturbing the visibility of the fluid. To remove the stickiness behaviour of the fluid we have used reducing agents such as HCL and NaOH. 5 Molar NaOH solution was found to give best results. The NaOH solution only etches the oxide layer and does not hamper the Galinstan or the glass tube. We put a few milliliters of NaOH on the top of Galinstan in the tank. Because of its low density, a small meniscus of NaOH is always maintained above the Galinstan column. The weight of this meniscus is negligible, and it is transparent and hence nearly invisible.

### Device operation

**Figure 3:**
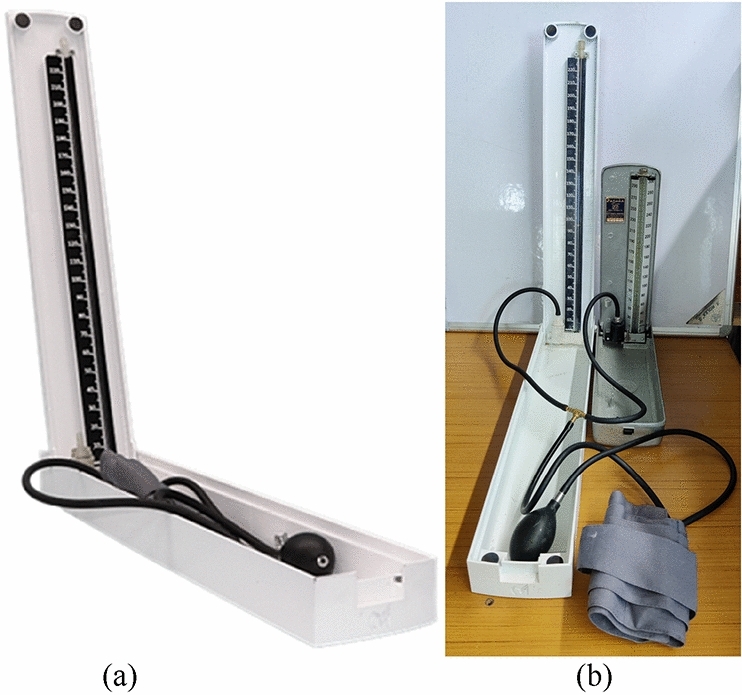
(**a**) Complete alpha-prototype of Merkfree, (**b**) technical comparison of Merkfree asainst MS through a T-connector joint.

The look, structure, and feel of the device remain the same as the conventional MS. Auscultatory method is used to measure BP using this device as with the MS. The prototype of the device is shown in Fig. 3a. The Galinstan has the same silvery color as mercury. Thus, there is no significant change in visualizing the fluid across the tube^26^.

### Ethics approval

This study was conducted in accordance with relevant guidelines, regulations and approved by the Institutional Ethics Committee (IEC:2021-654) of Dayanand Medical College Ludhiana, India. Written informed consent was obtained from all the participants of the study.

## Clinical trial

To compare the performance of the proposed Merkfree sphygmomanometer against commonly used sphygmomanometers in hospitals, we performed clinical trials after ethical clearance from the Institute ethical committee (IEC) of Dayanand Medical College and Hospital, Ludhiana. All methods were performed in accordance with the relevant guidelines and regulations. All the measurements were performed by trained clinicians Fig. 3b. A total of 252 participants, across various age groups (12–80 years, mean ± standard deviation = 41.23 ± 15.56 years) and sex (143 male, 109 female), were tested for systolic blood pressure (SBP) and the diastolic blood pressure (DBP) measurements**.** Informed consent was obtained from all subjects and/or their legal guardians**.** We took comparative measurement with the Merkfree and compared these with the commonly used MS (BPMR-120 Mercurial BP Delux from Diamond company) and a validated digital oscillometric device (WatchBP Office, Microlife).

### Technical validation study protocol

To check the technical accuracy of pressure readings of Merkfree, we compared Merkfree pressure readings against pressure readings of MS by connecting them to each other. For this, the air tube from cuff was split into two using a T-connector, one tube goes to MS and the other to Merkfree, ensuring equal pressure build up in both devices. Cuff was removed.

### Clinical study protocol

There were two teams appointed to measure BP. Each team had three members assigned to take readings. A 10-min time interval was given for every successive measurement from the same subject. Separate sheets were provided to each medical staff to avoid any correlation or bias due to the previously taken readings. The measurement order was first by MS followed by Merkfree and digital oscillometric device.

## Results

### Results from technical validations

**Figure 4:**
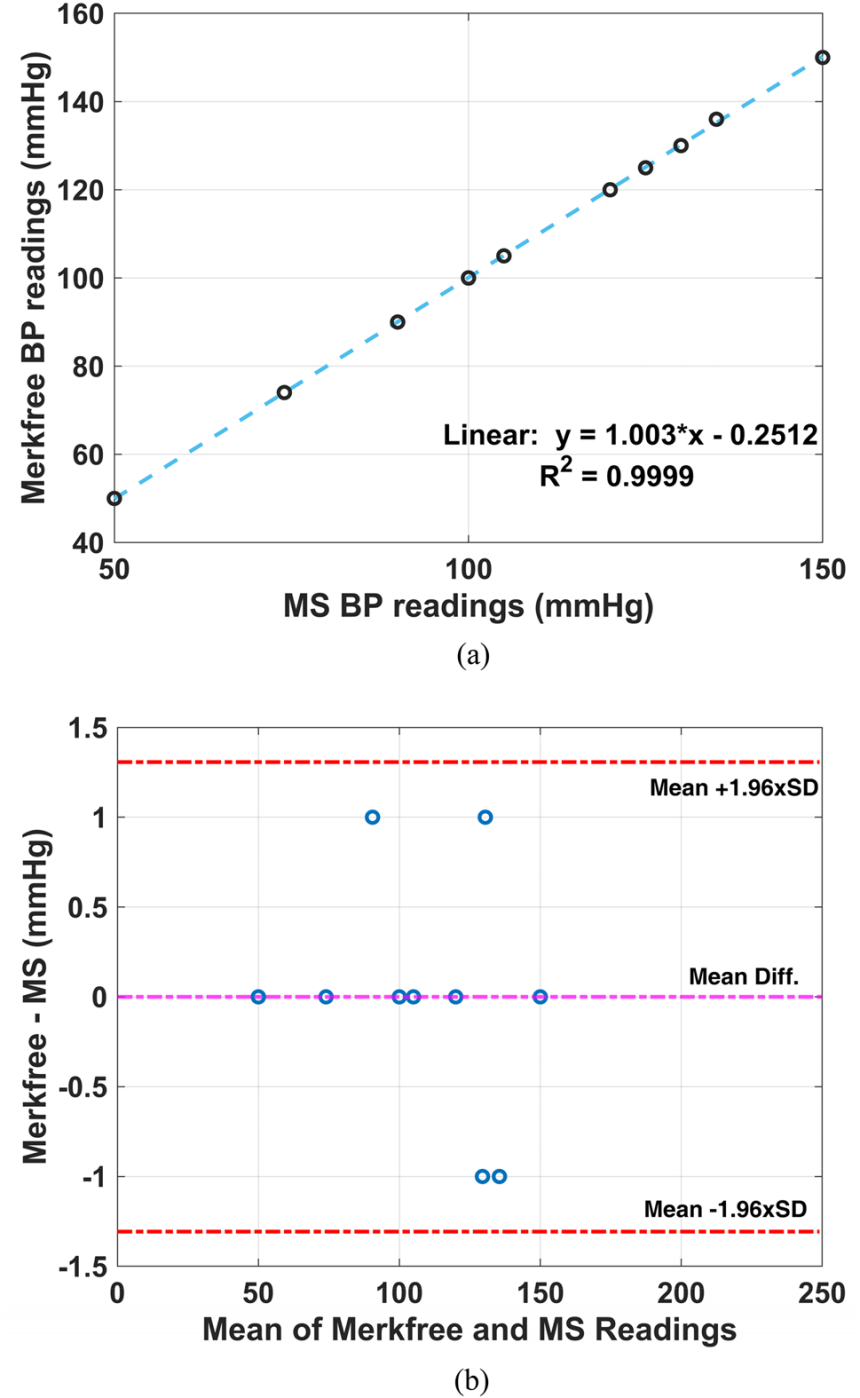
Comparison of pressure readings of Merkfree and Mercury sphygmomanometer. (**a**) The corresponding correlation values, (**b**) Bland Altman’s plot.

In this Merkfree and MS, were compared with each other directly through a T-connector joint as explained in section III(*B*). We took the data spanning wide pressures ranging from 50 to 150 mmHg. Results are presented in Fig. 4a. The pressure readings from two sphygmomanometers are almost equal, with a correlation value of 0.9999. The Bland–Altman plot, reports the bias value of 0 mmHg with standard deviation.

(SD) of mere 0.67 mmHg, among the two values. All the pressure readings are well inside the upper (1.3067) and lower (− 1.3067) limit-of-agreement (LOA) (bias ± 1.96 × SD), as clear from Fig. 4b. Moreover, the maximum difference between the readings of the two sphygmomanometers is a mere 1 mmHg, which may be attributed to parallax or human errors associated with BP measurements. Both sphygmomanometers have least count of 1 mmHg, rendering the differences depicted here negligible.

### Clinical comparison of Merkfree amd MS

**Figure 5:**
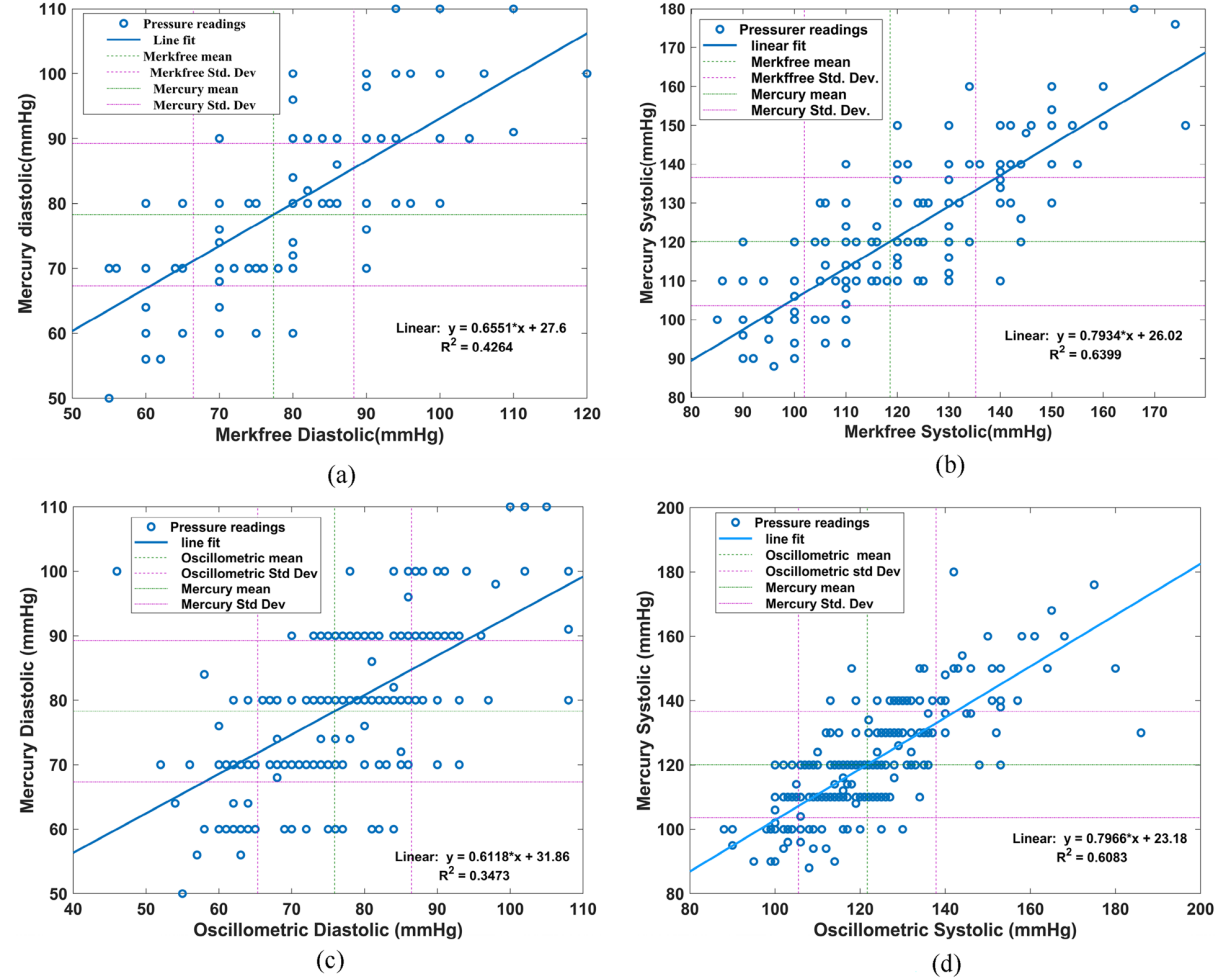
Comparison of blood pressure readings: (**a**) DBP readings of Mercury versus Merkfree, (**b**) SBP readings of Mercury versus Merkfree, (**c**) DBP readings of Mercury versus Oscillometric, (**d**) SBP readings of Mercury versus Oscillometric.

**Figure 6:**
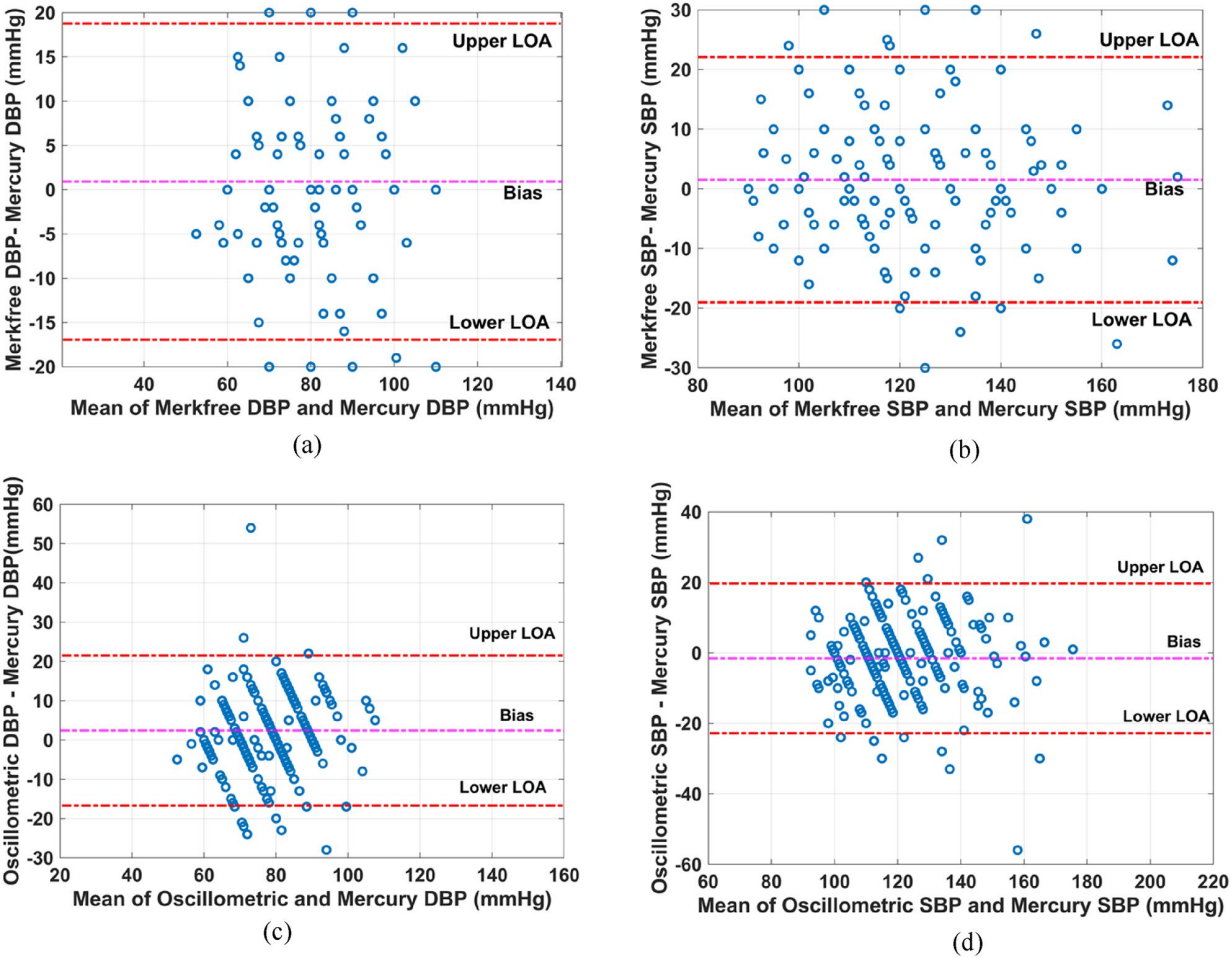
Bland Altman’s plot (**a**) DBP readings of Merkfree versus Mercury, (**b**) SBP readings of Merkfree versus Mercury, (**c**) DBP readings of Oscillometric versus Mercury, (**d**) SBP readings of Oscillometric versus Mercury.

The BP measurements done using Merkfree and MS have a coefficient of determination (R^2^) of 0.6399 for SBP and 0.4264 for DBP measurements; shown in Fig. 5a,b, respectively. The corresponding p-values of the correlation factor for SBP is 0.0216 and 0.115 for DBP. Bland–Altman’s analysis comparing Merkfree, and MS reveals that BP measurements have a bias of 1.528 mmHg with SD value of 10.49 mmHg for SBP Fig. 6a, and 0.916 mmHg with SD of 9.112 mmHg for DBP measurements Fig. 6b. The upper and lower limits of agreement (LOA) were calculated to be 22.09 mmHg and − 19.03 mmHg for SBP, and 18.78 mmHg and − 16.94 mmHg for DBP, respectively. Additionally, the mean absolute percentage error between the measurements from two sphygmomanometers is computed to be 6.22% for SBP and 8.6433% for DBP measurements.

### Clinical comparison of OS and MS

To understand the level of agreement between MS and OS, a comparison analysis was done. The BP measurements correlate with a R^2^ value of 0.6083 for SBP and 0.3473 for DBP measurements, as shown in Fig. 5c,d, respectively. Bland–Altman’s analysis shows that bias between the readings taken using these two sphygmomanometers is − 1.563 mmHg with SD value of 10.85 mmHg for SBP Fig. 6c and 2.401 mmHg with SD of 9.753 mmHg for DBP measurements Fig. 6d. The upper and lower LOA were computed to be 19.69 mmHg and − 22.82 mmHg for SBP and 21.52 mmHg and − 16.71 mmHg for DBP, respectively. In addition, the mean absolute percentage error between the measurements from two sphygmomanometers is computed to be 6.72% for SBP and 9.84% for DBP measurements.

### Clinical comparison of Merkfree and OS

Finally, a comparison of readings from Merkfree and OS was performed. The R^2^ for SBP and DBP were 0.62 and 0.41 respectively. The mean absolute percentage error between measurements from two sphygmomanometers is 7.015% for SBP and 8.94% for DBP measurements.

## Discussion

BP measurements using the proposed Merkfree sphygmomanometer has good agreement with commonly used MS and OS. Assuming that MS readings are the true readings of BP, in terms of percentage error, Merkfree readings have lower error compared to OS readings for both SBP and DBP. Merkfree readings also have a higher correlation with MS as compared to correlation obtained between OS and MS. Merkree SBP has lower agreement with OS SBP than MS SBP. Interestingly, this reverses in case of DBP, and Merkfree DBP has higher an agreement with OS SBP than MS SBP.

Merkfree shows comparable performance with respect to the gold standard MS during direct one-to-one comparison of pressure readings in technical validation using a T-connector joint. The Merkfree has a 0.9999 correlation factor with MS in one-to-one direct comparison in technical validation. The bland Altman’s analysis also reveals that bias is zero, which corresponds to the zero-measurement error, all data points lie within the limit of agreements and shows the good agreement between pressure readings of two devices. However, the same results were not reproduced during clinical trials, which can also be associated with the accumulation of various errors, such as hearing and concentration variation among individuals while measuring BP, white coat hypertension, and patient anxiety^27^.

**Figure 7:**
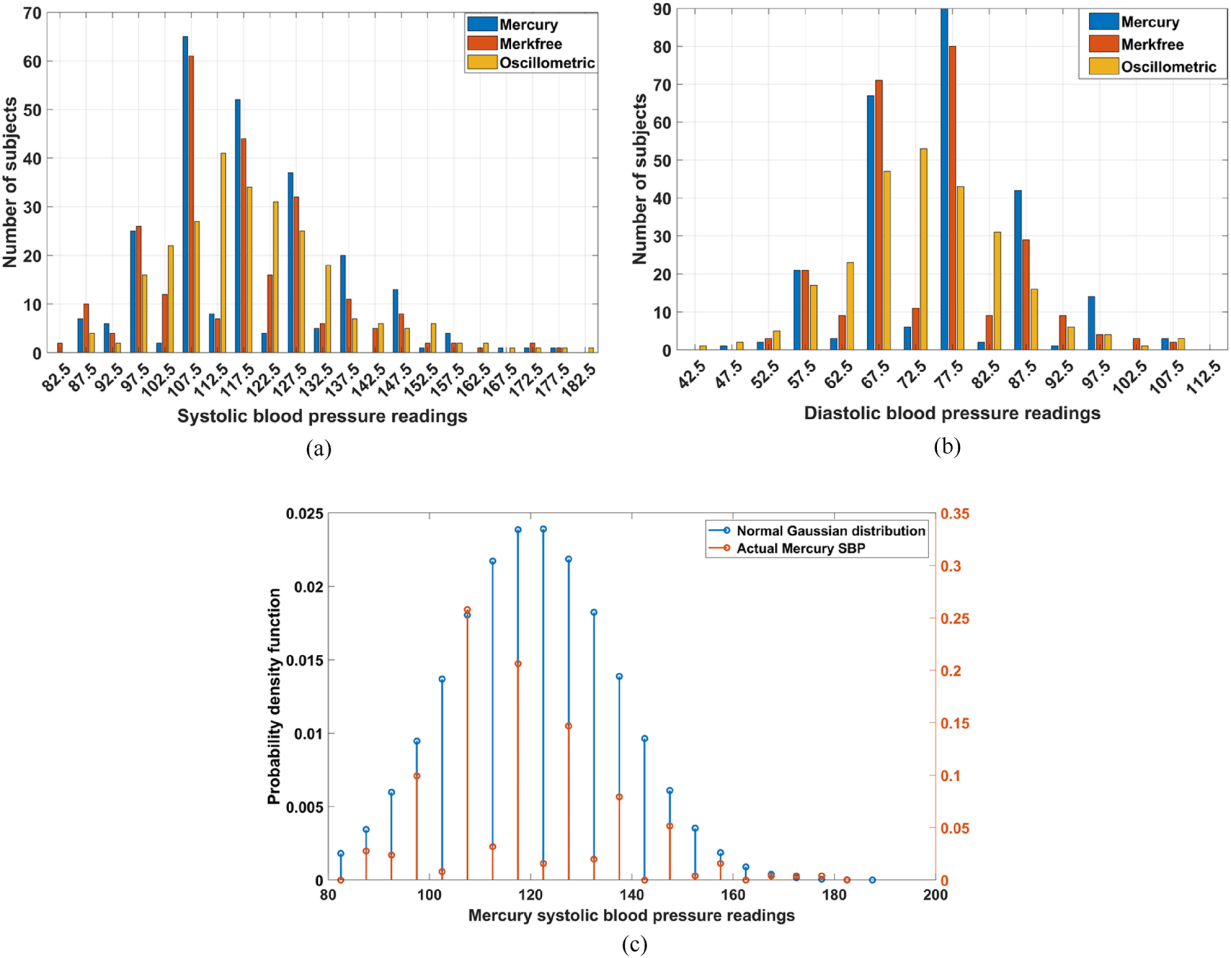
(**a**) Histogram plot of three devices for SBP, (**b**) Histogram plot of three devices for DBP, (**c**) A plot showing normalized ideal expected gaussian distribution plotted over frequency distribution obtained clinical study for the particular case of MS SBP. These two series are used to obtain the KL divergence for MS SBP. Similar series is obtained for all other measurements and to ultimately obtain the values in Table 1.

Bar graphs for SBP and DBP for both all three devices were plotted by taking a frequency interval of 5 Fig. 7a,b. A bar at x represents the number of readings lying between x−2.5 to x+2.5, where x is the multiple of five. Three bars are bunched together at every multiple of 5 for a side-by-side comparison of distribution of readings from all three devices. All three devices show a broad normal distribution which is expected for a random population study like this. It is well known that MS suffers from rounding-off errors due to operator bias towards rounding the readings to the nearest multiple of 10^28^. This specifically happens as the mercury sphygmomanometers scale has major calibration tick marks at multiples of 10. This can be clearly visualized by comparing at the histogram bar heights at multiples of 10 and multiples of 5 in Fig. 7a,b. We can see that frequency of readings are in general higher at multiples of 10 than at multiples of 5. In case of Merkfree, we have a longer scale and calibration marks at multiples of 5. Hence, we see that the bias towards rounding off to nearest multiple of 10 is reduced and we have comparable heights of frequency bars even at multiples of 5. OS type doesn’t have rounding-off error at all because human in the loop is eliminated. Distribution of the yellow bars in Fig. 7a,b demonstrate this quite clearly.

To quantify the extent of the mismatch in the distribution with respect to the expected normal distribution, we created the theoretically expected distribution and calculated its KL divergence with respect to the expected distribution. We have SBP and DBP data for all three devices which are 6 variables in total. For each variable X, we found the mean and standard deviation. An ideal gaussian distribution was calculated for MS SBP Fig. 7c using just the mean and the standard deviation. This distribution was then sampled at multiples of 5 starting from 85 to 195 mmHg for SBP; and from 45 to 125 mmHg for DBP. These ranges cover all readings starting from lowest to the highest in all columns. KL Divergence was then calculated between this distribution and the actual distribution for all 6 variables. The results are listed in Table 1. Here we can see that MS has highest KL divergence while OS has lowest indicated that MS have highest deviation from Normal distribution, primarily owing to rounding-off errors while OS has no rounding-off errors. Merkfree while maintaining better accuracy than OS, is also having lower rounding-off error compared to MS. This is apparent from the intermediate KL divergence values.

**Table 1:** KL divergence between ideal and actual distribution of readings for SBP and DBP of all three devices.

Column name	KL-divergence value
MS SBP	0.4088
MS DBP	0.4756
Merkfree SBP	0.1991
Merkfree DBP	0.2581
OS SBP	0.0848
OS DBP	0.0423

Most developed countries have already banned the MS, and most developing countries are in the process of doing so^29^. Merkfree is likely to be widely applicable and acceptable in these changed circumstances where MSs are discouraged. Digital BP measuring devices are independent of the operator, but their accuracy and repeatability have been questionable due to variability in proprietary software algorithms from company to company. As per the study conducted by us recently among Indian Clinicians^13^, it was found that digital BP measuring devices are considered inferior in accuracy and robustness compared to auscultatory BP monitors by most respondents. Owing to auscultatory method and longer measurement scale, Merkfree is expected to have limited use in home monitoring of BP. However, it is expected to have wide acceptance for clinical measurement of BP.

## Conclusion

We have designed a mercury-free sphygmomanometer Merkfree, with the same structure and principle of operation as a MS. Merkfree aims at being very close to the MS while getting rid of the mercury. Merkfree will help in achieving the goal of the Minamata convention of WHO by promoting elimination of Mercury based sphygmomanometers. Merkfree measures BP using same principle of auscultation as that of MS and was demonstrated to have less than 10% error with respect to MS for both SBP and DBP. Key innovation in Merkfree is the use of Galinstan instead of Mercury. Galinstan has a lower density compared to Mercury hence, Merkfree has a longer scale height. However, this improves visibility and reduces rounding-off errors. The stickiness of Galinstan with glass had been a major impediment in development of a BP monitor using it. This has been eliminated by developing an innovative technique of maintaining a small meniscus of a reducing agent in the measurement column. In this paper, we elucidated the different components of Merkfree device and conducted its clinical trials on 252 patients with respect to MS and OS.

## Data Availability

All data support our published claims and comply with field standards. The clinical trial data that support the findings of the study will be available from the corresponding authors subject to a reasonable request.
